# Florid Lobular Carcinoma In Situ: Imaging Characteristics and Pathologic Upgrade Rates on Surgical Excision

**DOI:** 10.1155/tbj/3580992

**Published:** 2025-03-30

**Authors:** Anshumi Desai, Susan B. Kesmodel, Barbara Susnik, Neha Goel, Yara Feliciano, Carmen Gomez-Fernandez, Youley Tjendra

**Affiliations:** ^1^DeWitt Daughtry Family Department of Surgery, Division of Surgical Oncology Miami, University of Miami, Miami, Florida, USA; ^2^DeWitt Daughtry Family Department of Surgery, Sylvester Comprehensive Cancer Center, Miami, Florida, USA; ^3^Baptist Hospital of Miami, Department of Pathology, Baptist Health System, Miami, Florida, USA; ^4^Department of Radiology, University of Miami, Miami, Florida, USA; ^5^Department of Pathology and Laboratory Medicine, University of Miami, Miami, Florida, USA

**Keywords:** florid lobular carcinoma in situ, invasive lobular carcinoma, lobular carcinoma in situ, upgrade rates

## Abstract

**Background:** Florid lobular carcinoma in situ is an uncommon lobular neoplasia variant that is frequently associated with invasive carcinoma. However, there remains a paucity of information to guide management. The authors aimed to study imaging features associated with pathologic upgrade rates for patients with florid lobular carcinoma in situ identified on core biopsy undergoing surgical excision.

**Methods:** Patients with florid lobular carcinoma in situ on core biopsy were selected from an institutional pathology database. Patients were excluded if pleomorphic lobular carcinoma in situ was also present on core biopsy. Clinical, radiologic, and pathologic features for each case were reviewed focusing on imaging features which led to core biopsy and those associated with pathologic upgrade on surgical excision.

**Results:** Eighteen cases of florid lobular carcinoma in situ underwent surgical excision. Upgrade rates on surgical excision were higher in cases with suspicious calcifications (8/11, 73%, *p*=0.049) compared to those without (1/7, 14.3%) and in cases with larger breast lesions (*p*=0.011). The overall upgrade rate was 50% (9/18), 89% (8/9) with invasive lobular carcinoma and 11% (1/9) with ductal carcinoma in situ. Of the 8 cases with upgrade to invasive lobular carcinoma, 7/8 (87.5%) were Stage I cancers and only 1/8 (12.5%) had macroscopic lymph node involvement and was upgraded to Stage II.

**Conclusion:** Florid lobular carcinoma in situ on core biopsy had an upgrade rate on surgical excision of 50% overall, with 89% of these cases upgraded to invasive lobular carcinoma. Pathologic upgrade was seen more frequently with suspicious calcifications and larger breast lesions. These findings can help guide surgical management of this uncommon lobular neoplasia variant including planning extent of excision and consideration for lymph node evaluation.

## 1. Introduction

The term “lobular carcinoma in situ” (LCIS) was coined by Foote and Stewart in 1941 [1]. Over the years, several authors have proposed different terminologies [2, 3]. Based on the current World Health Organization (WHO), LCIS is the preferred terminology and is defined as a noninvasive neoplastic proliferation of dyscohesive cells that originate from the terminal duct lobular unit (TDLU) and is characterized by an absence of E-cadherin expression [4–6]. Lobular neoplasia encompasses atypical lobular hyperplasia (ALH), classic LCIS (CLCIS), florid LCIS (FLCIS), and pleomorphic LCIS (PLCIS) [5]. While ALH and CLCIS are recognized for their premalignant potential, other LCIS variants have been recognized as high-grade lesions with greater malignant potential [7]. Although the cytologic features of FLCIS remain similar to CLCIS, FLCIS is characterized by marked distention of acini or ducts (approximately 40–50 cells in diameter) or with little to no intervening stroma [5]. Necrosis is not required for the diagnosis but is often present, either as focal, single-cell, or comedonecrosis, akin to that observed in solid-type ductal carcinoma in situ (DCIS).

LCIS was initially thought to be a direct precursor to invasive lobular carcinoma (ILC), and mastectomy was recommended for management [8]. However, subsequent data showed that LCIS is a nonobligate precursor and marker for an increased risk of breast cancer in both breasts [8, 9]. According to the current National Comprehensive Cancer Network (NCCN) guidelines and American Society of Breast Surgeons consensus guidelines, surgical excision is recommended when FLCIS is diagnosed on core biopsy (CB) [10, 11]. Despite these developments, comprehensive knowledge about the clinical outcomes and management strategies for FLCIS remains incomplete [12]. The authors aimed to study imaging features associated with pathologic upgrade rates for patients with FLCIS identified on CB and undergoing SE to help guide clinicians in the management of this entity.

## 2. Materials and Methods

### 2.1. Study Design

This is a retrospective observational study that was conducted at the University of Miami and associated hospitals. Cases with FLCIS diagnosed on CB were identified from the institutional pathology database from 2012 to 2023.

#### 2.1.1. Inclusion Criteria

• Patient age ≥ 18 years.
• Diagnosis of FLCIS on CB.

#### 2.1.2. Exclusion Criteria

• Presence of invasive carcinoma or DCIS within the same quadrant.
• Presence of PLCIS in the CB sample.

Patient demographic and treatment data were extracted from the electronic medical record. Imaging features, including the imaging target that led to the recommendation for CB and target size, were collected and reviewed by a fellowship-trained breast radiologist (Y.F.). The amount of tissue sampled was assessed by comparing the images taken before the biopsy and the images of the sampled tissue. All pathology slides were reviewed by three fellowship-trained breast pathologists (B.S., C.G.F., and Y.T.). Histopathologic features of FLCIS on CB and SE were documented. This study was approved by the Institutional Review Board as part of an ongoing assessment of quality and management for pathology and surgical oncology cases.

### 2.2. Technique of Core Biopsy

Under sterile technique, the breast was anesthetized and a small incision was made in the skin. A vacuum-assisted device or a spring-loaded device was inserted through the incision. Mammogram, magnetic resonance imaging (MRI), or ultrasound images confirmed the needle position in pre- and postbiopsy stages. Multiple cores were obtained. Subsequent specimen digital radiograph demonstrated the presence of the lesion within the cores. A localizing marker was placed through the biopsy probe and confirmed with postbiopsy images.

### 2.3. Technique of Surgical Excision

For patients undergoing partial mastectomy, the target lesion was preoperatively localized with a wire or other localization device by the radiologist under imaging guidance. Under anesthesia, the surgeon removed the lesion with the help of the localization device, and an intraoperative specimen radiograph was performed to confirm removal of the target lesion with a rim of normal breast tissue. The specimen radiograph was reviewed by the surgeon and the radiologist intraoperatively to confirm adequate removal of the target lesion. For patients undergoing surgical excision with mastectomy, standard mastectomy techniques were utilized.

### 2.4. Diagnostic Criteria

WHO recognizes FLCIS as a distinct entity within the spectrum of lobular neoplasia [5]. FLCIS is characterized by marked distention of the TDLUs by monomorphic cells with cytomorphological features similar to CLCIS, typically at least 40–50 cells in diameter or with minimal to no intervening stroma between the distended acini [4–6]. While FLCIS can be associated with necrosis and calcifications, these features are not required for diagnosis. In our study, we used these criteria to classify FLCIS cases, and we used E-cadherin immunostaining to confirm the diagnosis and distinguish it from ductal proliferations. A retrospective search of the pathology database was performed to identify cases of lobular neoplasia that were not classified as CLCIS or PLCIS. A subset of these cases, diagnosed before the introduction of the FLCIS terminology by the WHO in 2019, was originally classified as lobular intraepithelial neoplasia grade 3 (LIN3) with or without necrosis and/or calcifications or as a lobular neoplasia variant and was reclassified as FLCIS. Overall, all selected cases were in concordance with the original diagnosis.

### 2.5. Analysis

Descriptive statistics were used to summarize imaging and pathology characteristics. An unpaired *t*-test was used for continuous variables (size of imaging abnormality). The Fisher's exact test was utilized to compare categorical variables. A *p* value < 0.05 was considered statistically significant.

## 3. Results

### 3.1. Patient Characteristics

A total of 20 cases of FLCIS were identified in 19 patients. One patient was diagnosed with FLCIS on CB twice, with the biopsies performed in two different years and in two different quadrants of the breast. All patients were female. The median age was 57 years (range 35–77 years [SD 9.8]). Of the cases, 15 (75%) were in white women and 5 (25%) in black women, while 11 (55%) were identified as Hispanic and 9 (45%) as non-Hispanic. Surgical excision (SE) was performed in 18 cases with 50% (9/18) associated with an upgrade to ILC or DCIS. Two patients were managed with active surveillance and endocrine therapy without SE and were excluded from additional analysis.

### 3.2. Imaging Characteristics

**Figure 1:**
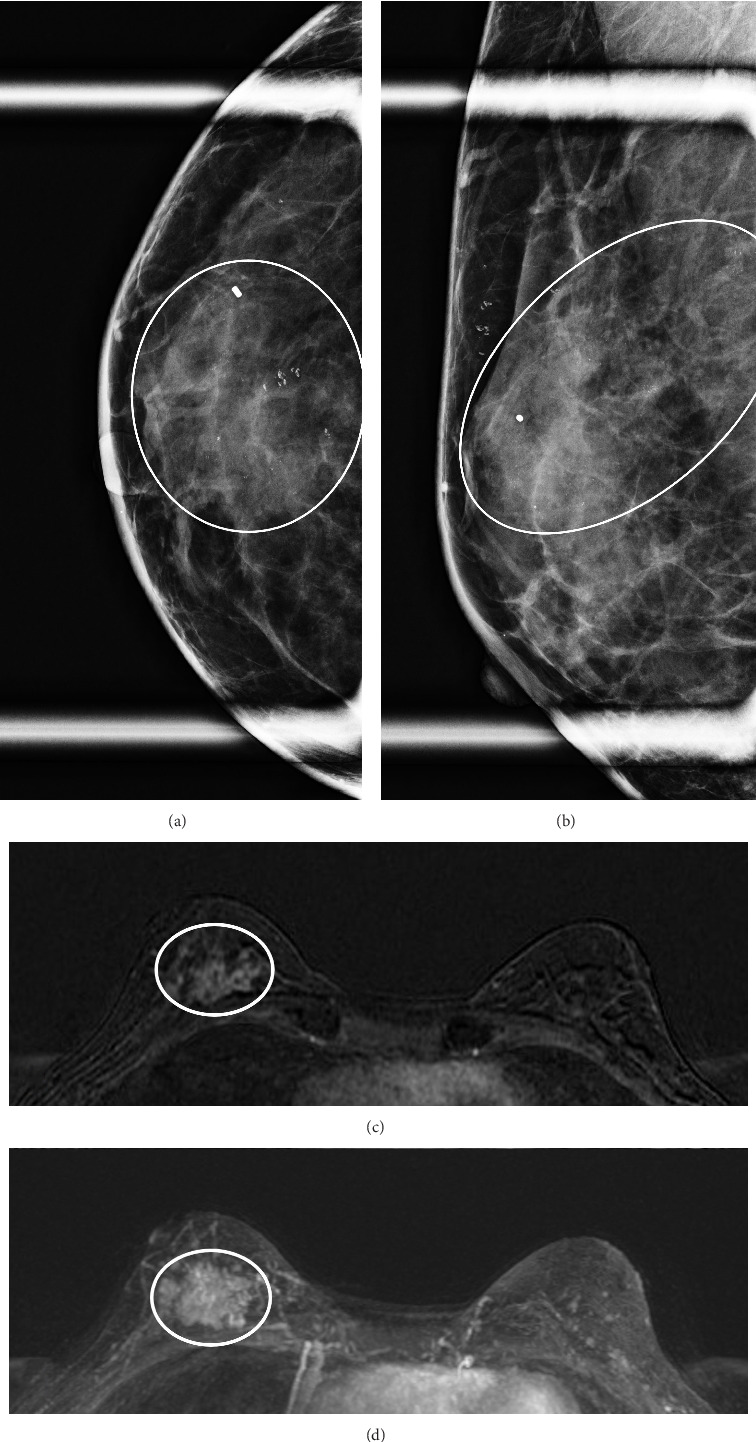
(a and b) Right breast magnification CC and ML views show segmentally distributed coarse heterogeneous calcifications. (c and d) Breast MRI T1 subtraction and maximum intensity projection axial sequences show regionally distributed clumped nonmass enhancement in the right breast.

Table 1 shows the imaging features of patients who underwent SE with and without pathologic upgrade, including biopsy modality and target. The most common biopsy modality was stereotactic (10/18, 56%), with equal numbers of patients undergoing biopsy by ultrasound (4/18, 22%) and MRI (4/18, 22%). The most frequent indications for biopsy were suspicious calcifications alone (8/18, 44%) and mass ± calcifications (5/18, 28%). Overall, suspicious calcifications, either alone or in conjunction with other imaging features, were present in 61% (11/18) of cases. Of these, 73% (8/11) were associated with an upgrade on SE. Cases with the presence of any calcifications on breast imaging had a higher upgrade rate on SE (*p*=0.049). Overall, 78% (14/18) of cases were biopsied with a 9-gauge needle, and the size of the sampling needle was not associated with upgrade. An example of imaging from a patient who was diagnosed with FLCIS with an upgrade to ILC on SE is shown in Figures 1(a), 1(b), 1(c), and 1(d).

The lesions were further divided by the size of the imaging targets as ≤ 10 mm, > 10 mm to ≤ 20 mm, and > 20 mm. Six cases were assigned to each size category (6/18, 33% each). In cases with an imaging target of ≤ 10 mm (6/18, 33%), only 1/6 (17%) had an upgrade on SE, while in cases with an imaging target of > 10 mm to ≤ 20 mm (6/18, 33%), 2/6 (33%) had an upgrade on SE. In contrast, in those cases with an imaging target of > 20 mm (6/18, 33%), pathologic upgrade on SE was identified in all 6/6 (100%) cases (*p*=0.009). The mean size of the target lesion for nonupgraded cases was 0.94 cm, while it was 4.66 cm for the cases with an upgrade. Overall, larger lesions identified on breast imaging had higher upgrade rates on SE (*p*=0.011). When a smaller amount of the lesion was sampled/removed on CB, upgrade rates were also higher on SE (*p*=0.049).

### 3.3. Pathologic Features

**Figure 2:**
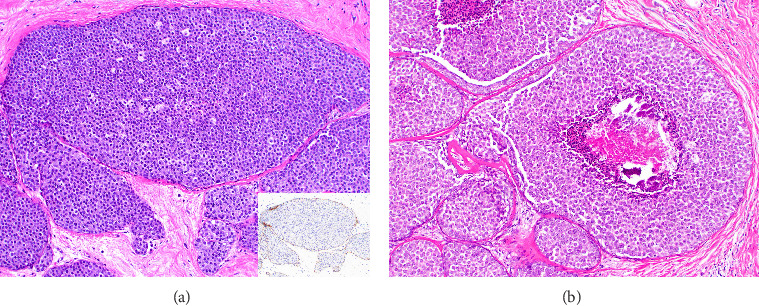
(a) Histologic section of core biopsy shows marked expansion of terminal duct lobular units with discohesive monomorphic cells (hematoxylin and eosin [H&E], 20x), negative for E-cadherin by immunohistochemistry (inset). (b) Florid lobular carcinoma in situ with associated comedonecrosis and calcifications (H&E, 20x).

Table 2 summarizes the histopathologic characteristics of FLCIS on the CB. On CB, FLCIS was associated with comedonecrosis (Figure 2) in 13/18 (72%) cases: 6/9 (67%) cases without upgrade and 7/9 (78%) cases with an upgrade. FLCIS with comedonecrosis was associated with upgrade on SE in 7/13 (54%) cases, while only 2/5 (40%) cases without comedonecrosis were upgraded on SE. Of the 11 cases with suspicious calcifications on imaging, with or without other imaging findings, 8/11 (73%) were upgraded on SE. Most of the cases of FLCIS were strongly ER-positive, 6/9 (67%) nonupgraded cases, and 9/9 (100%) upgraded cases. Among cases without upgrade, 2/9 (22%) had no residual FLCIS and 7/9 (78%) had FLCIS with or without background CLCIS on SE (Table 2). Among upgraded cases on SE, 1/9 (11%) cases showed DCIS, intermediate nuclear grade and 8/9 (89%) cases were ILC. Most of the invasive upgrades (7/8, 87.5%) were Stage I breast cancers with only one case (1/8, 12.5%) with macroscopic lymph node involvement upgraded to Stage II. The overall upgrade rate was 50% (9/18), and the upgrade to ILC was 44% (8/18). For nonupgraded cases, clear margins on SE were obtained in 8/9 (89%) cases with FLCIS on the margin in 1/9 (11%) cases. In the 9 cases with an upgrade on SE, margins were negative for invasive cancer and DCIS in all cases. There were 2 (22%) patients who had FLCIS at the margin, and one patient was taken back to the operating room for mastectomy. There was no association between the different histopathologic characteristics and higher upgrade rates on SE.

### 3.4. Axillary Management

The authors further evaluated the axillary management of the 8 cases with ILC on SE. Five cases had a SLNB at the time of primary surgery as 4 of them underwent mastectomy and one had a concerning area on imaging. Two patients were taken back for SLNB, and one patient did not have any additional surgery due to the presence of a locally advanced cancer in the contralateral breast.

## 4. Discussion

ALH and LCIS are high-risk breast lesions that involve the clonal growth of distinct, noncohesive cells that expand and fill the TDLU. The distinction between ALH and LCIS is determined by the percentage of the TDLU affected with lesions classified as LCIS if they distend over 50% of the TDLU [9]. Controversy has surrounded the management of LCIS since it was first described [13]. Historical studies have demonstrated that LCIS is a marker of increased risk of developing invasive ductal or lobular carcinoma; this risk is bilateral and persists beyond 20 years [3, 14]. LCIS is associated with a 1%–2% per year absolute risk of breast cancer with long-term follow-up after diagnosis. While CLCIS is a nonobligate precursor to ILC, the risk of progression to ILC is low [15]. FLCIS and PLCIS, however, are more aggressive LCIS variants that are more likely to progress to invasive carcinoma. Considering that DCIS is acknowledged as a precursor for invasive ductal carcinoma, the question arises as to whether high-grade LCIS variants such as FLCIS should be similarly treated [16]. However, the American Joint Commission of Cancer (AJCC) staging manual currently does not include FLCIS and PLCIS as part of the staging [17]. Although the NCCN currently recommends complete surgical excision for FLCIS, the optimal margin width is not defined [11].

Several studies have examined imaging features and upgrade rates associated with LCIS variants, although none have specifically focused on the correlation between imaging findings and upgrade rates in FLCIS. CLCIS is usually mammographically occult and is frequently identified on CB or SE for other imaging targets [18–22]. On the other hand, LCIS variants such as FLCIS and PLCIS are typically associated with imaging findings that can be targeted for biopsy [23, 24]. In this study, the most common biopsy target was suspicious calcifications alone, 44% of cases. Overall suspicious calcifications, either alone or in conjunction with other imaging features, were present in 61% of cases. Kuba et al. showed similar imaging findings when they studied PLCIS and FLCIS, with mammographic calcifications being the most common biopsy target [12]. Georgian-Smith et al. also studied calcifications associated with LCIS and found that calcifications associated with high-grade LCIS were similar in appearance to DCIS calcifications on mammograms [25]. However, none of these studies evaluated the association of calcifications on imaging and upgrade rates on SE. In the current study, of the cases where calcifications were present on imaging, 73% were associated with a pathologic upgrade on SE, suggesting an association between the presence of calcifications in FLCIS and upgrade rates on SE.

In addition to imaging features, the authors also assessed the association between the imaging size of the breast lesion and upgrade on SE. For “in situ” lesions, radiological findings such as lesion size on imaging are often known to be associated with an upgrade or “invasiveness” [26]. Park et al. identified predictors of upgrade for DCIS and found that cases with a lesion size of less than 2 cm on mammography and MRI were less likely to be upgraded [27]. Similarly, a mass size of more than 1 cm was associated with upgrade rates when Mooney et al. performed a study for high-risk breast lesions [28]. However, there are no studies that discuss the size of the imaging lesion for LCIS, specifically for FLCIS and upgrade rates on SE. In this study population, the imaging target size of upgraded FLCIS was significantly larger than that without upgrade, and an upgrade was identified in all lesions greater than 20 mm in size. This implies that larger lesions are more likely to have pathologic upgrades on SE. This may be helpful when counseling patients regarding surgical management, particularly the extent of surgical excision and consideration for lymph node evaluation.

FLCIS is often associated with necrosis, but this histopathologic finding is not required for the diagnosis [5]. Kuba et al. reported in their series that 71% of cases of FLCIS had necrosis and 23.5% of these were upgraded [12]. The current study showed an upgrade on SE in 54% of FLCIS with comedonecrosis compared to 40% of FLCIS without comedonecrosis.

Because studies have shown that CLCIS has a lower likelihood of upgrade on SE and is associated with a long-term bilateral risk of breast cancer, management has shifted from SE to high-risk surveillance and risk-reducing strategies [9]. In a systematic review and meta-analysis of CLCIS, Shehata et al. showed a 5.8% overall upgrade rate and a 3.5% upgrade to invasive carcinoma [29]. In contrast, multiple small retrospective series have shown significantly higher upgrade rates on SE in the setting of FLCIS [21, 30]. Shin et al. reported 21 cases of carcinoma in situ that histologically resembled solid-type DCIS but lacked E-cadherin expression which was defined as FLCIS. Of these cases, 57% (12/21) were upgraded to invasive breast carcinoma, of which 83% (10/12) were ILC [31]. Sullivan et al. reviewed upgrade rates of LCIS variants where LCIS with necrosis was classified as FLCIS. The overall upgrade rate was 25% with a more frequent upgrade to invasive carcinoma seen with FLCIS (36%) compared to PLCIS (18%) [32]. It is important to distinguish FLCIS from CLCIS as FLCIS is associated with invasive breast cancer in 35%–67% of cases, and most of these invasive cancers are lobular [7, 16]. In the current study, a 50% upgrade rate was seen among cases that underwent SE after a CB diagnosis of FLCIS, with ILC present in 89% of upgraded cases and 44% of cases overall. Despite multiple such studies showing an increased upgrade with FLCIS, there is a lack of data on the appropriate management [20, 30, 32]. Knowing the high upgrade rates, the authors recommend complete SE of the imaging abnormality with negative pathologic margins. No cases were encountered where distinguishing FLCIS from CLCIS was challenging. According to current NCCN guidelines, there are no specific recommendations for addressing CLCIS at the surgical margins, while recommendations exist for FLCIS. This distinction is important because the management of CLCIS does not require achieving negative margins, whereas negative margins are recommended for FLCIS [11].

In the present study, all of the invasive cancers were early stage (87.5% Stage I) with macroscopic lymph node involvement in only one case (12.5% Stage II). Although the rate of lymph node involvement was low, information regarding lymph node involvement may still be important for guiding adjuvant systemic therapy recommendations particularly in premenopausal women and extent of adjuvant radiation therapy after lumpectomy and mastectomy. Therefore, SLNB should be discussed and considered at the time of surgery when mastectomy is performed or when the area to be excised is large and may preclude SLNB at a second surgery.

One of the major limitations of this analysis is that it is a single institutional, retrospective study that only included 18 cases of FLCIS followed by SE. This limited the ability to identify meaningful clinical features that were associated with higher upgrade rates on SE. Therefore, a larger number of patients are necessary to confirm the association between suspicious calcifications and target lesion size on imaging with upgrade rates on SE. However, the strength of the study is that the cases were managed by a multidisciplinary team of physicians with advanced training. Fellowship-trained breast pathologists and radiologists reviewed all cases in this study, applying careful pathologic–radiologic correlation. In addition, the SE excisions were primarily performed by surgeons with surgical oncology or breast fellowship training who are more familiar with high-grade variants of LCIS leading to appropriate consideration for excision and judicious use of SLNB.

## 5. Conclusion

FLCIS is an uncommon histopathologic diagnosis on CB and is associated with high upgrade rates to invasive carcinoma on SE. In the present study, suspicious calcifications and imaging target lesion size greater than 20 mm were associated with significantly higher upgrade rates on SE. Given the high upgrade rates and the potential for FLCIS to be a direct precursor to ILC, complete SE of these lesions with negative margins similar to DCIS is recommended. In addition, in cases where a mastectomy or a large excision is performed which may preclude axillary sampling later and information regarding lymph node involvement may be important for adjuvant therapy recommendations, SLNB should be considered. Larger multi-institutional studies that evaluate the correlation between imaging findings and pathologic surgical upgrade rates are necessary to confirm these findings.

### 5.1. Clinical Practice Points

FLCIS is an uncommon lobular neoplasia variant and is frequently associated with invasive carcinoma. Physicians should be vigilant for suspicious calcifications and larger lesions in FLCIS cases, on imaging in FLCIS cases, as these imaging findings may be associated with higher upgrade risks on SE. SLNB should be considered during mastectomy or in cases where extensive excision is required. Surgeons should aim for clear margins in SE akin to DCIS management given the high upgrade rates to invasive cancer. A multidisciplinary team of breast radiologists, pathologists, and surgeons is recommended for accurate diagnosis and management decisions.

## Figures and Tables

**Table 1:** Radiologic characteristics of FLCIS cases.

	No upgrade on SE (*n* = 9)	Upgrade on SE (*n* = 9)	*p* value
Biopsy modality			
Stereotactic guided	4 (44%)	6 (67%)	0.637
Ultrasound guided	2 (22%)	2 (22%)	1.000
MRI guided	3 (33%)	1 (11%)	0.576
Biopsy target			
Calcifications	3 (33%)	5 (56%)	0.637
Mass			
With suspicious calcifications	—	2 (22%)	0.471
Without suspicious calcifications	2 (22%)	1 (11%)	1.000
Asymmetry			
With suspicious calcifications	—	1 (11%)	1.000
Without suspicious calcifications	1 (11%)	—	1.00
NME	3 (33%)	—	0.205
Presence of any calcifications	3 (33%)	8 (88%)	0.049⁣^∗^
Size of the imaging target			
≤ 10 mm	5 (56%)	1 (11%)	0.131
> 10–20 mm	4 (44%)	2 (22%)	0.619
> 20 mm	—	6 (67%)	0.009⁣^∗^
Estimated tissue removed by CB			
< 50%	1 (11%)	6 (67%)	0.049⁣^∗^
50%–90%	4 (44%)	2 (22%)	0.619
> 90%	4 (44%)	1 (11%)	0.294
Needle size utilized for CB			
9 gauge	7 (78%)	7 (78%)	1.000
12 gauge	2 (22%)	—	0.471
14 gauge	—	2 (22%)	0.471

Abbreviations: CB, core biopsy; MRI, magnetic resonance imaging; NME, nonmass enhancement; SE, surgical excision.

⁣^∗^Statistically significant.

**Table 2:** Histopathologic characteristics of FLCIS on core biopsy and subsequent surgical excision.

	No upgrade on SE (*n* = 9)	Upgrade on SE (*n* = 9)	*p* value
FLCIS on core biopsy			
Necrosis			
Comedonecrosis	6 (67%)	7 (78%)	1.000
Single-cell necrosis	1 (11%)	—	1.000
No necrosis	2 (22%)	2 (22%)	1.000
ER status			
ER positive > 50%	6 (67%)	9 (100%)	0.206
ER positive < 50% or negative	3 (33%)	—	0.206
Surgical excision			
FLCIS ± CLCIS	7 (78%)	—	
No residual FLCIS/only CLCIS	2 (22%)	—	
DCIS	—	1 (11%)	
ILC, stage 1	—	7 (78%)	
T1mi/T1a N0/NX⁣^∗^		4 (57%)	
T1b N0		1 (14%)	
T1c N0/N1mi		2 (29%)	
ILC, stage 2	—	1 (11%)	
T1mi N1a		1 (100%)	

Abbreviations: CLCIS, classic lobular carcinoma in situ; DCIS, ductal carcinoma in situ; ER, estrogen receptor; FLCIS, florid lobular carcinoma in situ; ILC, invasive lobular carcinoma.

⁣^∗^One patient did not have lymph node sampling.

## Data Availability

The data that support the findings of this study are available upon request from the corresponding author (A.D.). The data are not publicly available as they contain information that could compromise the privacy of research participants.
